# No supplementary evidence of attention to a spatial cue when saccadic facilitation is absent

**DOI:** 10.1038/s41598-018-31633-w

**Published:** 2018-09-05

**Authors:** W. Joseph MacInnes, Roopali Bhatnagar

**Affiliations:** National Research University Higher School of Economics, Moscow, Russian Federation

## Abstract

Attending a location in space facilitates responses to targets at that location when the time between cue and target is short. Certain types of exogenous cues – such as sudden peripheral onsets – have been described as reflexive and automatic. Recent studies however, have been showing many cases where exogenous cues are less automatic than previously believed and do not always result in facilitation. A lack of the behavioral facilitation, however, does not automatically necessitate a lack of underlying attention to that location. We test exogenous cueing in two experiments where facilitation is and is not likely to be observed with saccadic responses. We also test alternate measures linked to the allocation of attention such as saccadic curvature, microsaccades and pupil size. As expected, we find early facilitation as measured by saccadic reaction time when CTOAs are predictable but not when they are randomized within a block. We find no impact of the cue on microsaccade direction for either experiment, and only a slight dip in the frequency of microsaccades after the cue. We do find that change in pupil size to the cue predicts the magnitude of the validity effect, but only in the experiment where facilitation was observed. In both experiments, we observed a tendency for saccadic curvature to deviate away from the cued location and this was stronger for early CTOAs and toward vertical targets. Overall, we find that only change in pupil size is consistent with observed facilitation. Saccadic curvature is influenced by the onset of the cue, buts its direction is indicative of oculomotor inhibition whether we see RT facilitation or not. Microsaccades were not diagnostic in either experiment. Finally, we see little to no evidence of attention at the cued location in any additional measures when facilitation of saccadic responses is absent.

## Introduction

Selective attention allows our visual system to preferentially process some information over others. Theories of attentional control often revolve around the dichotomy between top-down and bottom-up control processes, also described as endogenous and exogenous attention. Endogenous attention represents goal-driven processes^1–3^ while exogenous is guided by stimulus-properties^4–6^. Other factors have been suggested to supplement or modulate this dichotomy such as selection history^7^, associated reward^8,9^, context learning & memory^10^; task demands and complexity^11–13^, prior experience^14^; or a temporal continuum of top down and bottom up processes^15^.

Posner’s cueing paradigm^16^ has served as the backdrop in understanding spatial and temporal interaction of visual attention by adjusting cue/target properties to see how they affect responses to attended locations (mental chronometry). Typically, a location in space is cued with a peripheral onset or central arrow followed by a target at the cued or uncued location. Short cue-target onset asynchronies (CTOAs) will result in a facilitatory effect (faster RTs for cued targets) and longer CTOAs in an inhibitory effect termed inhibition of return (IOR: slower RTs for cued targets). The switch from facilitation to inhibition, is usually observed around a CTOA of 250–300 milliseconds (ms) though this may depend on task demands^17^. The facilitatory effect can be explained as an orientation of attention towards the cued location and improving further processing of the following target onset. At longer CTOAs, however, after visual attention is disengaged from the cued location facilitation gives way to inhibition of return^18^. Spatial cueing effects using predictive cues have been demonstrated in other species as well – monkeys^19^, rats^20^, honeybees^21^, archer fish^22,23^ highlighting their potential role in species survival. IOR is thus, seen as a ‘foraging facilitator’^24^ and has been suggested to improve search efficiency by reducing the likelihood of attention returning to already fixated locations^24–26^ (but see^27^). So, it may seem that looking at a relevant location twice may be part of human fixation selection strategy which is in fact a trade-off between foraging for novelty and fully understanding the relevant parts^28^.

Although facilitation from exogenous orienting is often described as reflexive and automatic, a number of studies have reported no facilitation at shorter CTOAs but instead early onset IOR^29,30^. Danziger and Kingstone^31^ for example, observe IOR within 50 ms at the cued location and Maruff *et al*.^32^ observed facilitation at short CTOAs but only if the cue and target overlapped temporally. Pratt, Hillis and Gold^33^ demonstrated the influence of spatial overlap and physical characteristics of stimuli on cueing effects by using three different types of cues. Out of the three experiment conditions, only one showed typical cueing effects while the others showed insignificant or zero facilitation at short CTOAs. Pratt, Sekuler and McAuliffe^34^ suggested an influence of attentional set on early facilitation. Taylor, Chan, Bennet and Pratt^35^ observed no facilitation and early IOR when potential target locations were not marked with placeholders. MacInnes^36^ tested the spatial and temporal gradient of IOR with continuous random CTOAs and also found no early facilitation for either manual or saccadic responses. Malevich, Ardasheva, Krueger and MacInnes^37^ tested the influence of temporal expectations on cueing effects and found no facilitation when the multiple CTOAs were random or mixed, but only observed facilitation when the CTOAs were blocked.

There seems little doubt that attentional set and top down expectation can modulate the appearance of facilitation, but what remains uncertain is whether attention was allocated to the cued location at all. The lack of behavioural facilitation of reaction time may reflect a complete lack of attentional capture, but it’s also possible that attention was removed too early to influence reaction times^37,38^ (RTs). Facilitation is not the only measure of attention in exogenous cuing, so we propose to explore alternate measures of attentional deployment.

## Alternative Measures of Attention Deployment

### Saccadic curvature

Although RTs have become a standard in measuring the deployment of spatial attention, a number of alternative methods have been proposed. Saccades to target locations are generally not straight, and the curvature deviation from a straight path has been shown to be influenced by covert attention^39,40^. Additionally, the strength of saccadic deviation reflects the amount of attention to a particular location as measured by target RTs. The trajectories of saccades deviating away from an attended location has been consistently seen in studies, but this effect does not translate to hand movements^41^.

The temporal aspects of saccadic deviations show the same biphasic pattern as reaction times over increasing CTOAs^42^. McSorley *et al*.^42^, reported that deviations away from a distractor were observed for longest latencies and deviations towards a distractor in case of shorter latencies with the transition point around latency of 200 ms. The same, however, does not hold for anti-saccades and longest latencies did not correspond to greatest distractor caused deviations^43^. Saccadic deviations are also influenced by the distance of the distractor to the target^44,45^, vertical distance of the distractor from the fixation^46^ and the target hemifield^47^.

Also, similar to reaction times, curvature deviations may change based on prior knowledge about the task^48^. In scenarios where target locations were known or predictable, saccade trajectories were found to be deviating away from the distractors and scenarios where target locations were unpredictable, saccades curved towards distractors.

### Microsaccades

The human visual system has been adapted to detect motion and so any stationary, unchanging scene would cause perceptual fading as the retina adapts to it. To counter this effect, oculomotor system generates micro movements (drifts, tremors and microsaccades) during a fixation. Microsaccades are fixational eye movements that are involuntary and ballistic with an average rate of 1–3 per second, magnitude of 12 to 15 minutes of arc and a typical duration of less than 10 ms (but see^49^ for an overview and why these sizes have been increasing). Microsaccades and saccades seem to be kinematically similar, existing on a functional continuum, implicating similar neural circuitry^50^. It has been shown that microsaccades occur not just during fixation but also during search and exploratory tasks^51^.

Recent results suggest that microsaccades are modulated by visual attention in spatial cueing paradigms^52–54^. Engbert and Kliegl^52^ reported that microsaccades tend to be biased towards the cued location in a spatial cueing task, but many other studies have shown microsaccade bias both towards and away from cue direction^53,55–57^. An interaction with the cue type has also been noted – endogenous attentional cues biasing microsaccade direction towards the cue, as governed by attentional shifts^58–60^ and exogenous attentional cues biasing microsaccade direction away from the cue, as per IOR^55^. Attentional cues also affect microsaccade rate^56,61^ as does task difficulty^60^. This has lead Laubrock *et al*.^62^ to propose that both microsaccade direction and RTs are strong indicators of spatial attention (but see^63^). Interestingly, microsaccades show biphasic modulation; that is, at stimulus onset, microsaccade rate drops to zero immediately and then recovers. This is known as ‘microsaccadic inhibition’^52,64^ and is interpreted as a top down effect on microsaccades to modulate sensory signal quality.

### Pupil size

Pupil size changes are a result of the interaction of the parasympathetic and sympathetic components of the autonomic nervous systems (ANS). The primary pupillary function being regulation of light entering the eye, resulting in pupillary light reflex (PLR). Pupil dilations have been noted due to factors other than luminance changes, like individual differences, cognitive load^65,66^, emotions^67^, attention^68^ and stimulus probability^69^, along with color perception and faces.

Pupil size, on average, is about 3 mm, which can increase by more than double (approx. 120%) due to change in illumination, but only by 0.5 mm due to cognitive factors^70^. Koss^71^ suggests there is a strong link between the locus coeruleus norepinephrine (LC-NE) system and the pupillary response, hence a change in LC activation can be tracked through changes in pupil size. LC-NE neurons project to a large number of brain areas, especially areas associated with attention – superior colliculus, parietal cortex, pulvinar nucleus.

Gabay, Pertzov and Henik^72^, measured pupillary response in monkeys in localization and discrimination tasks and suggested a correlation between pupil size and IOR at cue onset. Mathôt and colleagues^73^, tie the PLR to modulations in covert attention and suggest that this may provide a measure of behavioral cueing effects.

### Proposal

Peripheral onsets do not guarantee the capture of attention as measured by reaction times to targets at the cued location. The lack of capture on this single measure, however, does not tell us to what degree the cue was attended, if at all. In the following two experiments, we compare predictable and continuous CTOA designs^36,37^ with a saccadic response and four possible target locations. Given our goal of testing alternate measures of attention when facilitation is absent, we tested CTOAs that were predictable within a block as a control condition that is likely to generate facilitation (E1) and a random CTOA distribution that has consistently failed to show facilitation effects at the cued location for both manual^37^ and saccadic^36^ responses (E2). We expect to observe robust IOR at mid to late CTOAs for both experiments, but expect no facilitation at early CTOAs with target validity when CTOA selection is chosen randomly within each block. With four possible equi-eccentric placeholders for cue and target (top, bottom, left and right), invalid target locations are possible at opposite and orthogonal to the cued location. This design allows us to test the impact of attention at valid and invalid spatial locations using a number of metrics not related to saccadic reaction time, i.e. saccadic trajectory, microsaccades and pupil size. Given that these measures have also been shown to be sensitive to attention, we expect our measures to converge in E1. If exogenous attention is present at the cued location despite a predicted lack of impact on SRTs in E2, it is possible that we will see its influence in microsaccades or pupil size at short CTOA, or in the saccadic curvature in trials where the cue is orthogonal to the target. If a lack of SRT capture means that attention is not allocated to the exogenous cue, we expect to see no impact of target validity on any of our saccadic metrics.

## Experiment 1: Blocked CTOA condition

### Methods

#### Participants

Twenty-five participants (11 males; age range 18–35 years; mean = 26 years) took part in the experiment. All participants reported normal or corrected-to-normal vision and no color blindness. Written informed consent was provided and an honorarium of 200 Rubles was given at the end of the session. The experiment was conducted with the approval of the Higher School of Economics (HSE) ethics board, and all methods were performed in accordance with the relevant guidelines and regulations. Although experiment one was conducted second, it is presented first to better frame the results of the second.

#### Stimuli and Apparatus

**Figure 1:**
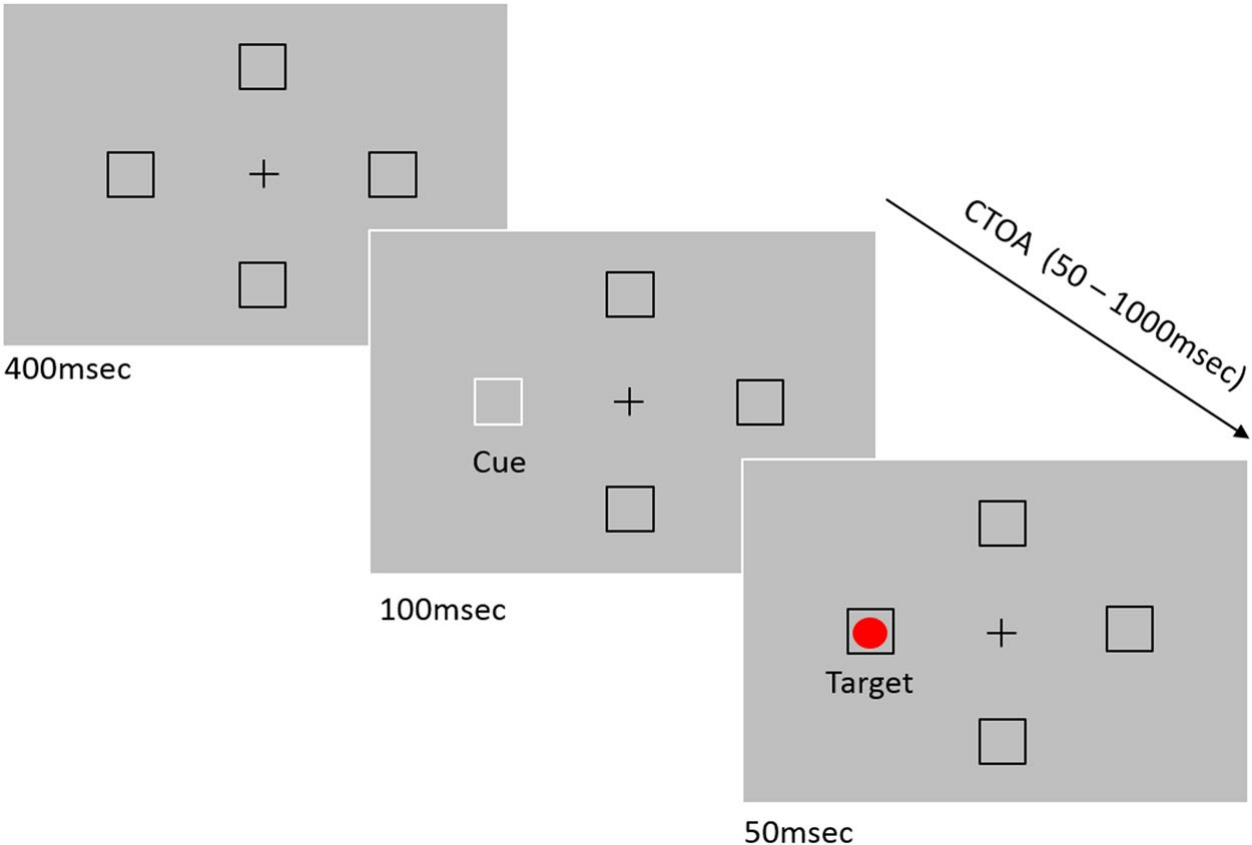
Illustration of a valid trial for 50 ms block. In the last slide representation, the top and bottom boxes are the orthogonal position (relative to target), the box on the right is opposite and the box with the target is valid. Orthogonal and Opposite may be combined as ‘Invalid’ locations; Valid and Opposite may be combined as ‘Parallel’ locations. The target shown in the horizontal hemifield.

Stimuli were presented on an ASUS VG248QE LCD monitor running at 120 Hz with a 1920 × 1080 pixels resolution and eye movements were recorded with SR-Research EyeLink II system (SR Research, Mississauga, Ontario, Canada) at a temporal resolution of 1000 Hz. Stimuli were generated using MATLAB (MathWorks, Natick, MA, USA) and Psychophysics Toolbox extension^74,75^. A five-point eye tracker calibration and validation procedure were done for each participant at the beginning of each trial block. The participant’s head was placed in a chinrest so that the eyes are at a distance of 80 cm from the screen. The stimuli were viewed binocularly, but eye movements from the right eye only, were analyzed. Stimuli and an experimental procedure are illustrated in Fig. 1. Each trial consisted of a fixation display with a gray background (CIE: 0.313, 0.329; 53.58 cd/m^2^), for a duration of 400 ms, showing a black (CIE: 0.307, 0.448; 0.66 cd/m^2^) central fixation cross and four black square placeholders (top, bottom, right, and left; 5° from fixation). The exogenous cue (white flash; CIE: 0.311,0.329; 99.85 cd/m^2^) appeared at any one of the four equi-eccentric locations (equal probability) for a duration of 100 ms and after an interval of 50 ms, 250 ms, 450 ms or 650 ms (blocked condition), the target (red dot; CIE: 0.611,0.329; 50 cd/m^2^) appeared randomly in any of the four locations for a duration of 50 ms.

#### Procedure

Participants were instructed to press the spacebar to commence the trial and drift correction, keep their eyes on the fixation, then wait for the red dot inside one of the placeholders and make an eye movement (saccade) to the target. The trial ended after this saccade and any error was signaled by a beep and a message on the display screen. The fixation display was presented for a duration of 400 ms, after which the cue flashed for 100 ms at any one of the placeholders. After a fixed-blocked CTOA (50 ms, 250 ms, 450 ms or 650 ms), the target was presented until response (maximum 5000 ms). There were 96 such trials in each 10-minute block (total four blocks) for each participant.

We varied three main factors, within subjects: stimuli location - cue location and target location were randomly presented at any of the four locations; cue validity - resulting in three possible values (valid, invalid-opposite or invalid-orthogonal); and target hemifield (horizontal or vertical). We used blocked CTOA (50 ms, 250 ms, 450 ms or 650 ms) condition and randomly varied the order of blocks for each participant. The dependent measures were the saccadic reaction time (SRT in ms), saccadic curvature, microsaccades between presentation of cue and target, and pupil size changes to the cue (pre- to post- onset).

#### Data analysis

Anticipatory responses or RTs <100 ms (0.94%), keyboard press errors (0.74%), fixation errors (8%), outliers with RTs >3 SD from mean (2%) and trials with blinks (9%) were excluded. Hence, 21% trials excluded using these criteria.

**Figure 2:**
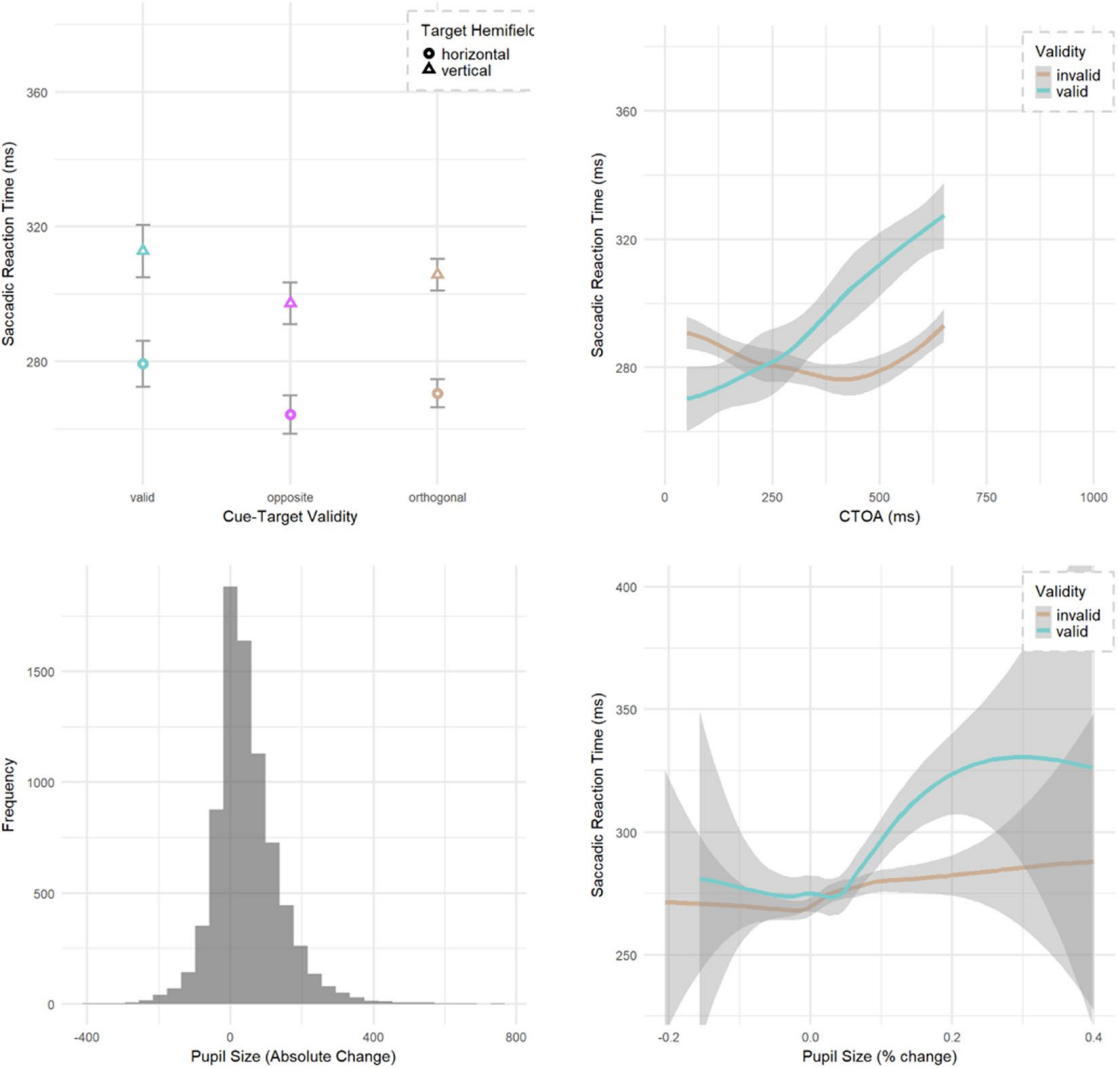
Interaction between cue validity and CTOA. Orthogonal and Opposite may be combined as ‘Invalid’; Valid and Opposite may be combined as ‘Parallel’. (**a**) Invalid trials did not differ significantly between the opposite and orthogonal location although the trend was for faster responses at the opposite location. (**b**) The valid and invalid RTs (ms) at each CTOA (ms), demonstrate the classic interaction of early facilitation and later IOR. (**c**) Post cue – Pre cue distribution of pupil area. (**d**) The relative size of the pupil helped predict SRT, with large changes suggesting slower valid trials.

Statistical analysis was done using the linear mixed effects (lme4) model^76–78^ in the R statistical package^79^. For the linear mixed effects model, we first defined a null model, with the participant as a random factors, and incrementally added fixed effects and random slopes (target hemifield, cue-target location, CTOA and pupil size change) to the model to see if their inclusion improved the model. We used the chi-squared (χ2) test to check if a new model was an improvement over the previous one. The model was tested for both slopes and intercepts, with by participant random slopes for CTOA improving the model fit (χ2 (2) = 295, p < 0.001), with all other fixed effects being intercepts. There was no difference between the two invalid trial options (opposite and orthogonal, t < 1.0: and see Fig. 2a) so these were combined for a single ‘invalid’ option for target location. Zero condition for all models was selected as invalid, horizontal hemifield and 50 ms CTOA.

### Results: E1

The mean model reaction time was 260.1 ms, standard error 6.9 ms (for the baseline condition of horizontal target hemifield, 50 ms CTOA, invalid trial) for the final model. The slope of the probe onset time was significant as nested within participant (χ2 (2) = 88, p < 0.001). For fixed effect intercepts, there was a significant main effect of target hemifield (χ2 (1) = 272, p < 0.001) with vertical locations having slower RTs (28.8 ms, SE 1.7 ms) than horizontal. There was a main effect of validity (χ2 (1) = 29, p < 0.001) with valid trials being faster than invalid (−19.6 ms, SE 3.4 ms) at the base of 50 ms CTOA, and this was qualified by a validity x CTOA interaction (χ2 (2) = 90, p < 0.001) with valid trials slowing (+8.4 ms SRT/100 ms CTOA, SE 1.1 ms) (Fig. 2b). We further see an interaction between the change in pupil diameter as a result of the cue and the cue validity (χ2 (2) = 12.4, p = 0.002). In effect, every 100 units of pupil size increase after the cue slowed responses (+5 ms/100 pupil size, SE 2.2) to validly cued locations only (Fig. 2d).

**Figure 3:**
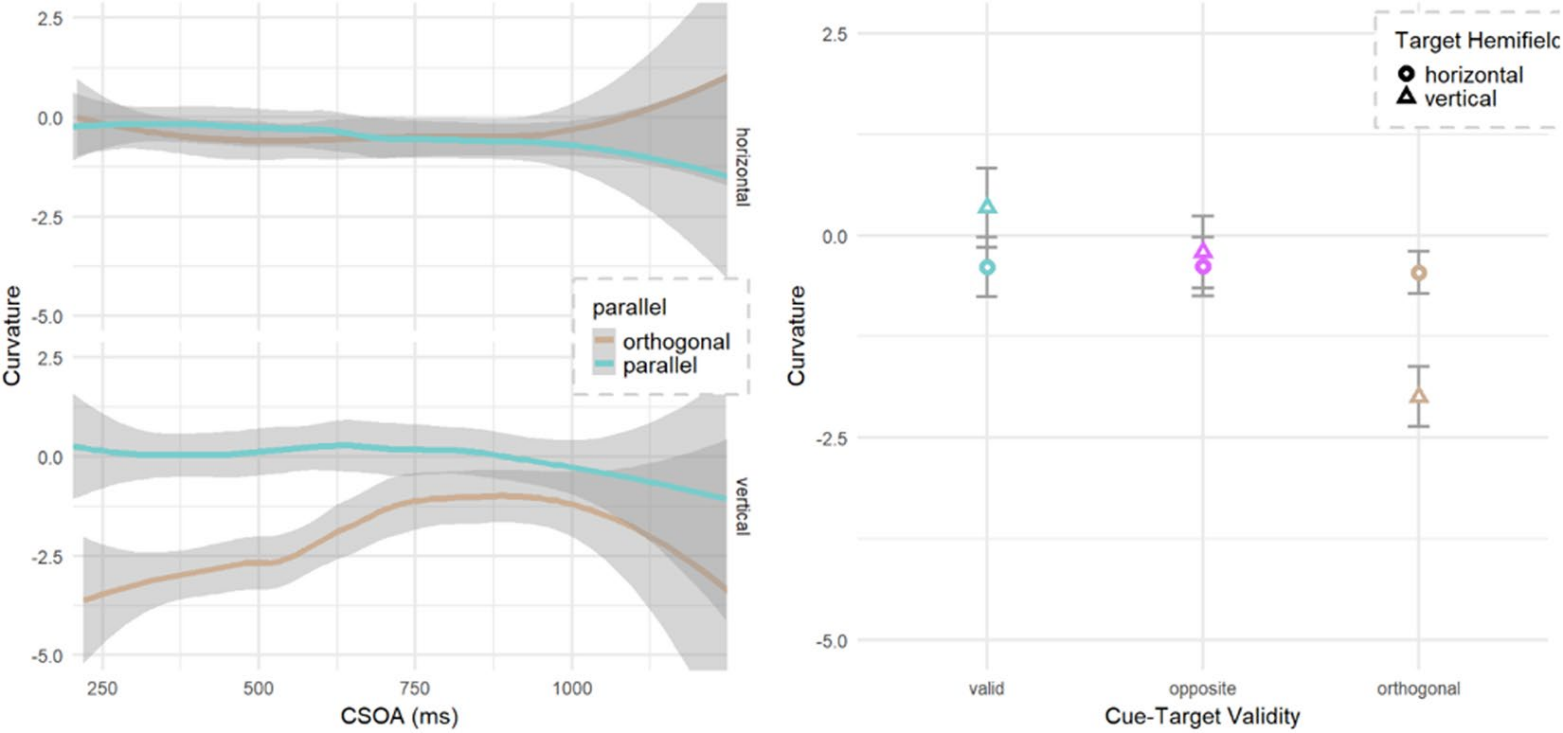
Saccadic curvature. Orthogonal and Opposite may be combined as ‘Invalid’; Valid and Opposite may be combined as ‘Parallel’. CSOA is the interval between the onset of the cue and the onset of the saccadic response. (**a**) Control condition of parallel (targets *valid* or *opposite* to the cue, merged together) show little bias in the CCW (negative) direction. (**b**) Saccadic curvature is biased away from the cued location when targets are orthogonal, and this effect is strongest for the vertical hemifield and at early cue-saccade onset asynchronies.

Analysis of saccadic curvature used the signed area under the curve with negative values reflecting curvature away from the cued location on orthogonal trials and counter clockwise (CCW) when the cue and target directions were either *valid or opposite* (*henceforth parallel*). Cues that were orthogonal to the target were further modified by changing their sign using the winding technique from computer graphics to determine if the curve was toward (+) or away from (−) the cue. Fixed effects were the same as the SRT analysis, except that CTOA was modified to include cue-to-saccade onset time and named here as CSOA. The base model had a slight negative bias of −0.22 of area (SE 0.49) representing a tendency for CCW curvatures when cues and targets are parallel. There was a main effect of relative cue-target location with targets orthogonal being more negative (χ2 (2) = 45.3, p < 0.001) meaning a curvature away from the cued location (−0.46, SE 0.57). This was stronger (−3.6, SE 0.61) in the vertical hemifield (Fig. 3a) as evidenced by the parallel by hemifield interaction (χ2 (2) = 54.7, p < 0.001). Finally, this hemifield interaction was further modified by CSOA (Fig. 3b) with the strongest vertical, parallel curvature observed at early CSOAs (χ2 (2) = 12.2, p = 0.002).

Microsaccade analyses focused on the impact of the cue on the rate and direction of microsaccades. Following the analyses of Laubrock *et al*.^56^, we look at the rate of microsaccades in the range of immediately prior to the cue (−250 ms) to the CTOA following the cue (50 ms or greater). Since our CTOAs were randomly selected between 50 and 1000 ms, we calculated microsaccade rate as a frequency of observed microsaccades over the number of trials that match or exceed that CTOA.

We do see a slight reduction in rate immediately after the cue, but this is not as severe as the dip reported in Laubrock *et al*.^56^. Also, we do not see a recovery and increase in the rate at 300 + ms. An LME analyses of the three temporal groups shows a main effect of group (χ2 (1) = 83, p < 0.001), with an initial drop in rate after the cue, but this rate drop continues in the 300–600 ms range instead of recovering as in Laubrock *et al*.^56^.

**Figure 4:**
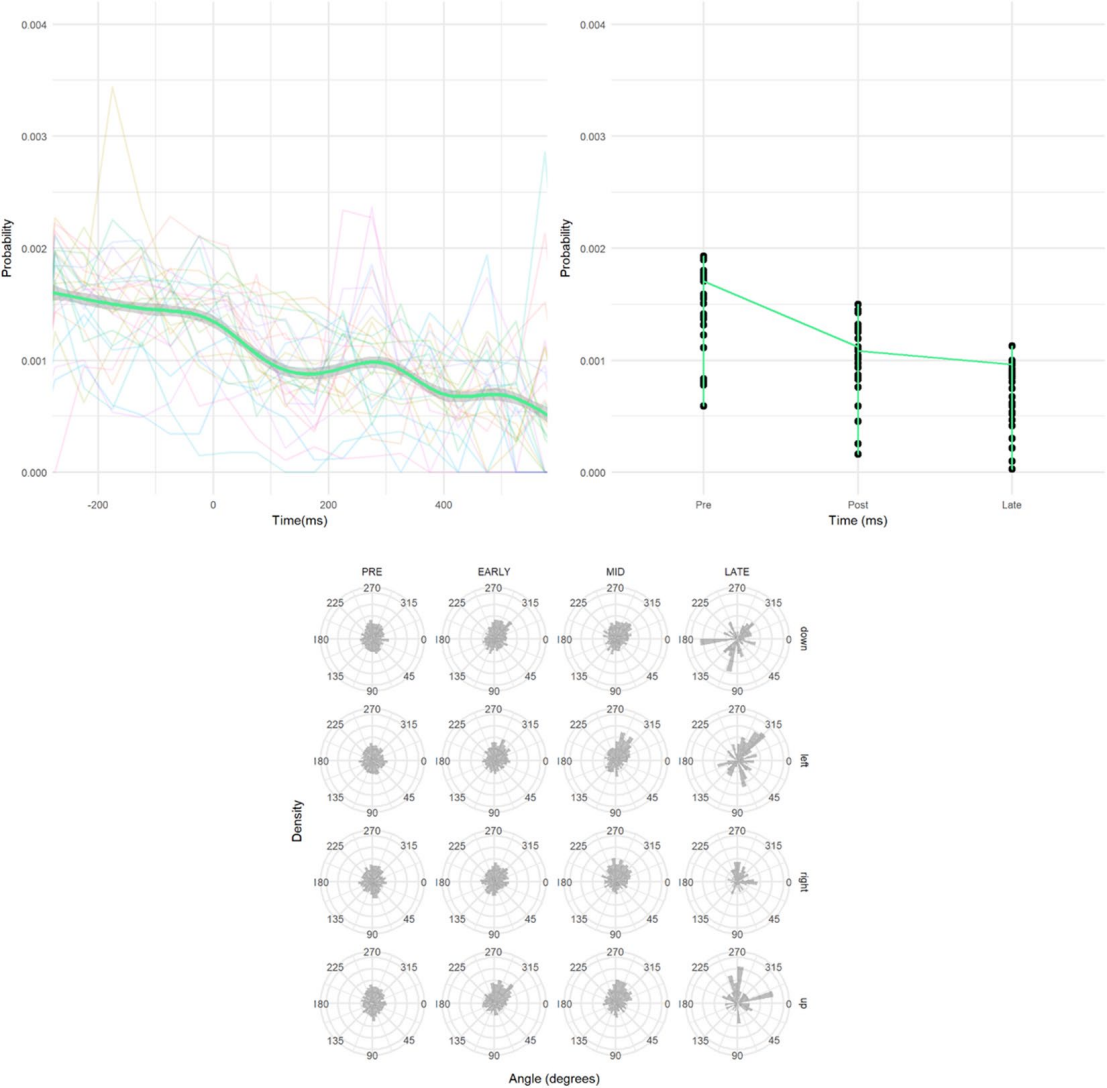
(**a**) Microsaccade rate plotted as the likelihood of a microsaccade at a given time compared to the cue onset with time = 0, representing the onset of the cue. There is a dip in microsaccade rate following the cue, but no recovery. (**b**) Microsaccade rate with probability prior to the cue, shortly after the cue and at longer intervals after the cue. (**c**) Directional bias of microsaccades at various time frames relative to cue. Pre-cue was up to 300 ms before the cue, Early, Mid and Late were up to 300, 600 and 900 ms bins after the cue respectively.

For microsaccadic direction, we again see no change in response to the cue, except perhaps at the latest time interval. Our results (Fig. 4) do not show the horizontal bias observed in Laubrock *et al*.^56^, though this is not surprising, since our display had vertical as well as horizontal locations. Also missing in our results is the early bias of microsaccades toward the cue and the later inhibitory bias away from the cue. An ANOVA of the total bias (microsaccades toward the cue minus total opposite the cue) for 100 ms bins before and after the cue shows a small effect of microsaccade time (F (10) < 1), unlike previous research, none of the bins differ significantly from 0.

### Discussion: E1

As predicted, we found significant facilitations at early (50 ms) CTOA. This SRT advantage was absent by 250 ms CTOA and became a cost at later CTOAs (450+) reflecting IOR at the cued location. In addition, we found that saccadic curvature was sensitive to the cued location with curvature away from the cue at early CTOA’s, especially for saccades toward vertical targets. We also observed that changes in pupil size help to predict SRT, with larger pupil changes reflecting slower RTs for targets at valid locations. Given that pupil size may suggest covert attention to the change in cue brightness^73^ we suggest both of these measures are consistent with trial by trial differences in attention allocated to the cue. Microsaccade rate and direction were also measured, but did not show any sensitivity to the cue onset or location. Given that two of our three additional measures showed sensitivity to the cue onset, we followed with a second experiment where we would not expect attentional capture as measured by SRTs. It is possible that these other measures could be more sensitive to exogenous allocation of attention and shed light on the question of whether attention may exist when facilitation is absent.

## Experiment 2: Random continuous CTOA

### Methods

The experiment design remains similar to Experiment 1, except the CTOA, which was a random interval varying from 50–1000 ms. Thirty participants (one excluded due to insufficient data; 14 males; age range 19–44 years; mean = 25 years) took part in the experiment. There were 368 such trials in a 45-minute session (with breaks) for each participant. Anticipatory responses or RTs <100 ms (4.13%), keyboard press errors (0.77%), fixation errors (7.30%), outliers with RTs >3 SD from mean (1.2%) and trials with blinks (1.09%) were excluded. Hence, 14.5% trials excluded using these criteria.

### Results: E2

**Figure 5:**
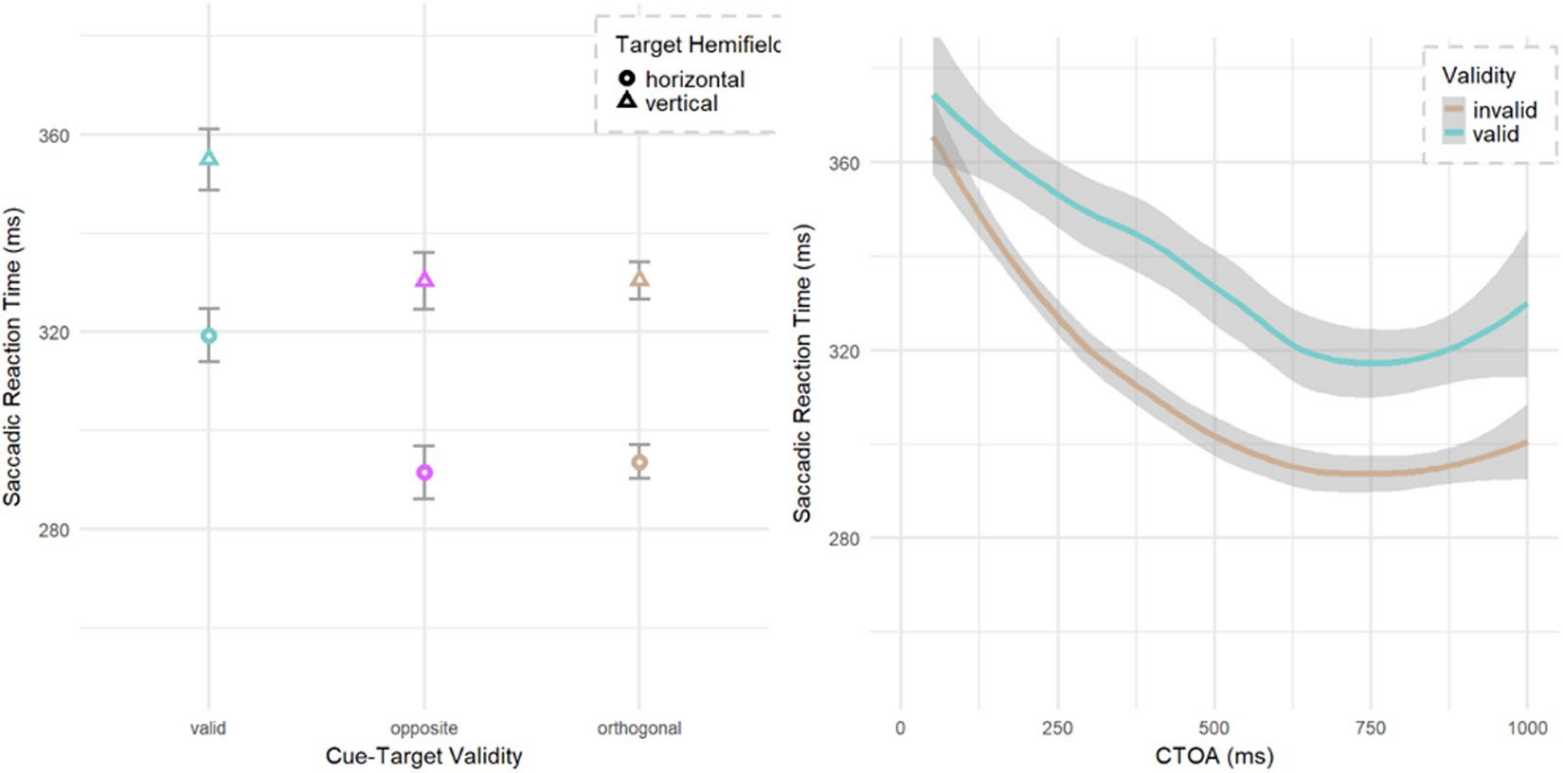
Main effect of IOR with valid trials slower than invalid trials. (**a**) Invalid trials did not differ between the opposite and orthogonal location. (**b**) The valid and invalid RTs (ms) at each CTOA (ms), demonstrating lack of facilitation and early onset IOR. Opposite and Orthogonal are combined as ‘invalid’.

The mean model reaction time was 323 ms, standard error 8.5 ms (for the baseline condition of horizontal target hemifield, 50 ms CTOA, invalid trial) for the final model. There was a significant main effect of cue validity (χ2 (1) = 148, p < 0.001) as validly cued locations had slower RTs (25 ms, SE 2 ms) than invalid trials (Fig. 5). This effect of IOR was significant from the outset of 50 ms with no validity by CTOA interaction (χ2 (1) = 0.02, p = 0.876, see also Fig. 2b). CTOA was also significant (χ2 (1) = 45, p < 0.001) with faster RTs (6.3 ms/100 ms CTOA, SE 0.6 ms) at late CTOAs. We observed strong significant effect (χ2 (1) = 397, p < 0.001) of target hemifield on the saccadic RT with saccades made in the vertical hemifield slower (37 ms, SE 2 ms) than those made in the horizontal hemifield.

We also tried to fit the change in pupil size as a fixed effect to see if there was a change in overall cue awareness as measured by pupil size. Although an interaction of pupil size and target hemifield does help predict overall SRT (χ2 (2) = 8.9, p = 0.011), pupil size does not interact with validity to differentially influence valid cues at any CTOA.

**Figure 6:**
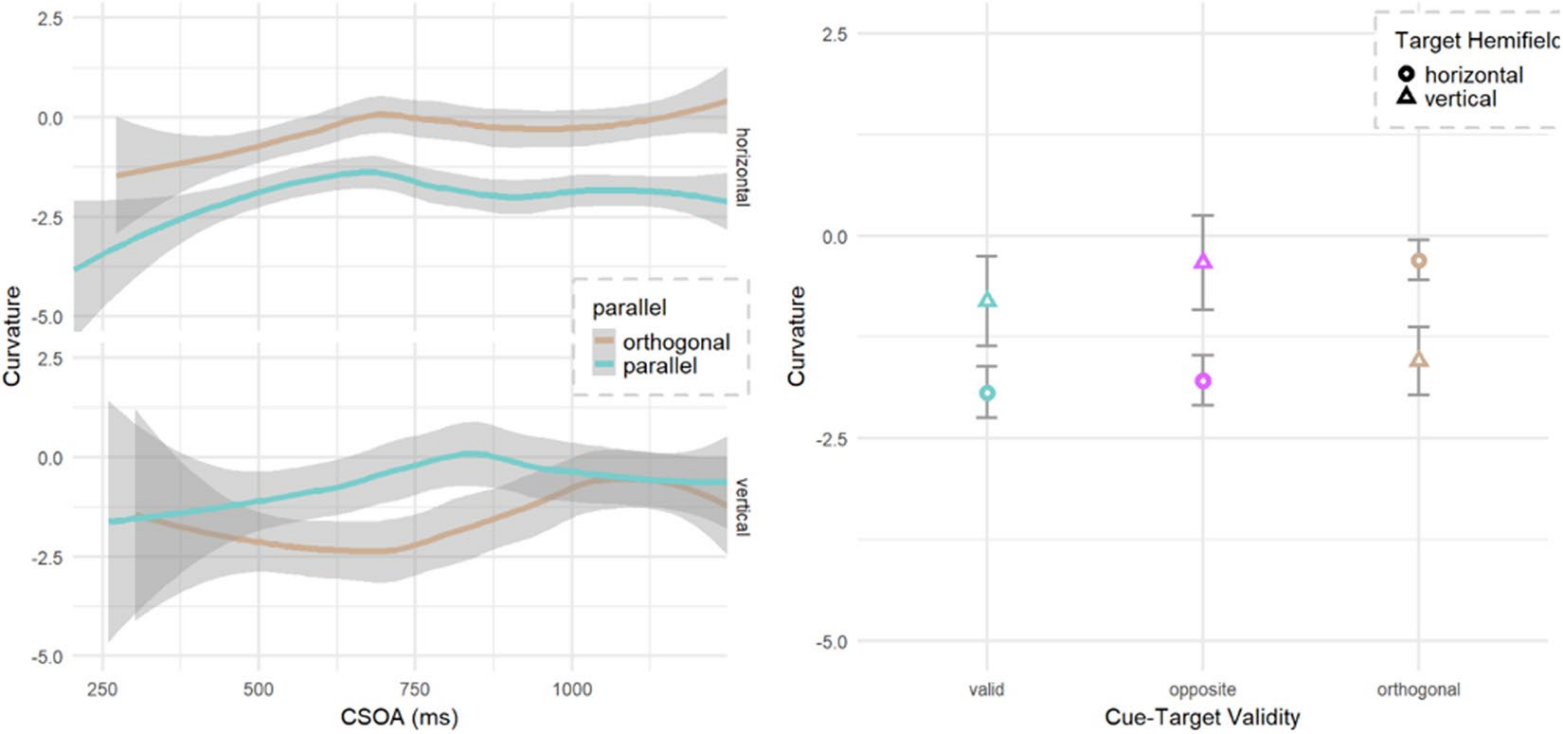
Saccadic curvature. Valid and Opposite are combined as ‘Parallel’. (**a**) Saccadic curvature results with negative curvatures reflecting a tendency away from the cued location for orthogonal trials and counterclockwise for valid and opposite trials. (**b**) Orthogonal cues invoked greater curvature toward the cued location at early CTOAs with reduced curvature bias at late CSOAs. Parallel (valid and opposite) trials showed a counter clockwise bias. While the hemifield did not significantly interact with CSOA nor relative cue location, they are plotted here since vertical saccades have often produced larger curvatures.

For saccadic curvature analyses, the LME was again tested for hemifield and relative cue-target location. CTOA was modified to include the saccadic latency (CSOA) to improve the model fit. Mean curvature for orthogonal, horizontal saccades was −1.14 normalized units of area under the curve (SE = 0.53) with negative tendency reflecting a curvature away from the cue. Relative cue-target location interacted with target hemifield to improve the fit of the model with the (χ2 (3) = 61.9, p < 0.001) with the control (parallel) curved more negative (toward counter-clockwise) than baseline (Fig. 6) but only for horizontal saccades. Orthogonal targets however, had a larger bias (away from the cue) for vertical targets. The addition of CSOA slope (χ2 (2) = 13.1, p = 0.001) and intercept (χ2 (1) = 18.1, p < 0.001) both improved the model fit with a reduction of the bias (see Fig. 6a) at later CSOAs (+1.0/second, SE = 0.44). No other effects or interactions were significant on saccadic curvature.

**Figure 7:**
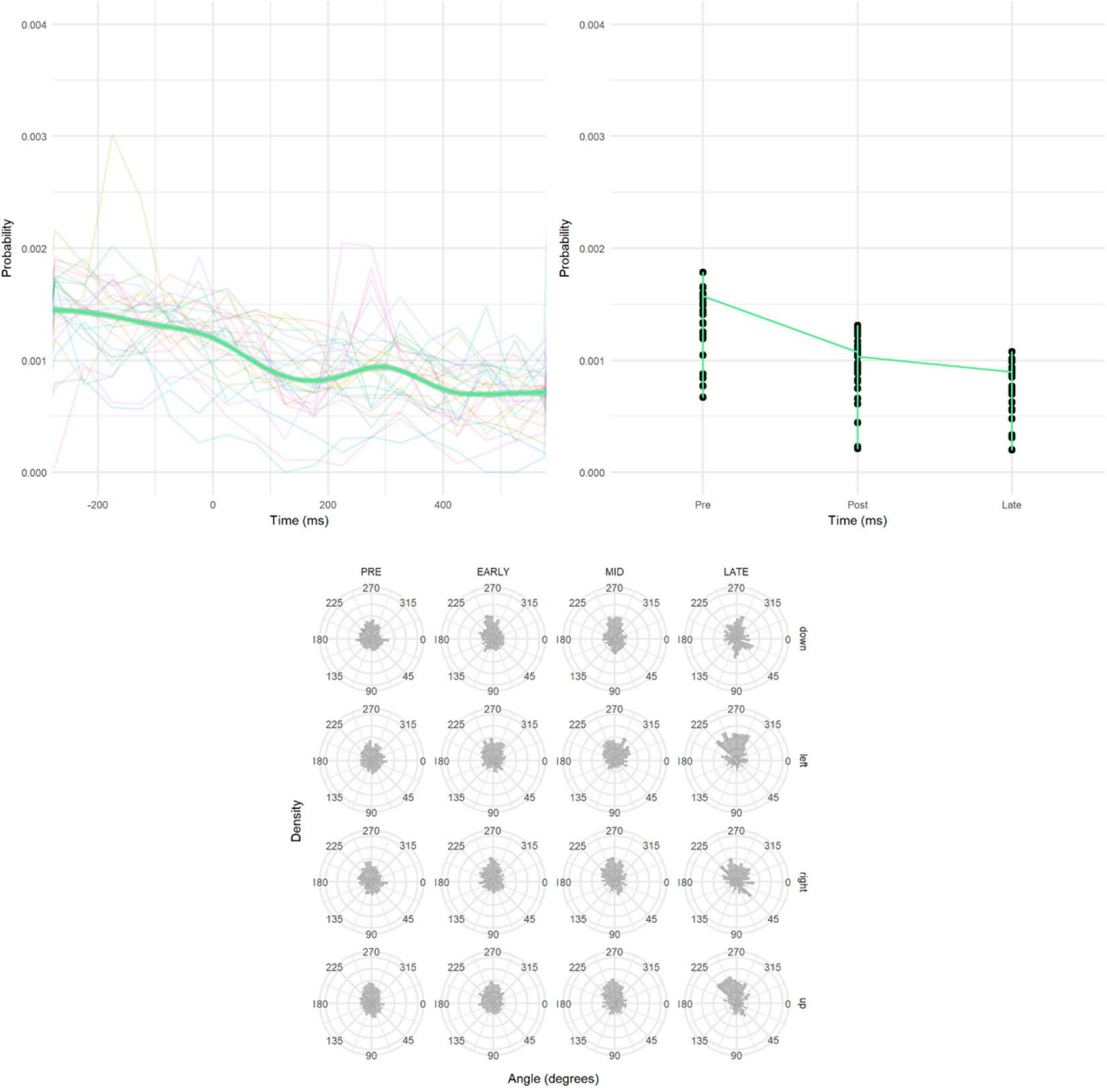
(**a**) Microsaccade rate plotted as the likelihood of a microsaccade at a given time compared to the cue onset with time = 0, representing the onset of the cue. We do see a dip in microsaccade rate following the cue, but not a recovery of the rate at 300 ms and later. (**b**) Microsaccade rate with microsaccade probability prior to the cue, shortly after the cue and at longer intervals after the cue. (**c**) Directional bias of microsaccades at various time frames relative to cue. Pre-cue was up to 300 ms before the cue, Early, Mid and Late were up to 300, 600 and 900 ms bins after the cue respectively.

Plotting the rate of microsaccades in the range of −300 ms to +600 ms as compared to the cue onset (Fig. 7a), we see a slight reduction in rate immediately after the cue, but this is not as severe as the dip reported in Laubrock *et al*.^56^. Also, we do not see a recovery and increase in the rate at +300 ms. An LME analyses of the three temporal groups shows a main effect of group (χ2 (1) = 70, p < 0.001), with an initial drop in rate after the cue, but this rate drop continues in the 300–600 ms range instead of recovering as in Laubrock *et al*.^56^.

For microsaccade direction, we performed a mixed effects regression to determine patterns of microsaccades towards the cued location prior to target onset. The dependent variables were a logit transform of the percentage of microsaccades toward the cued location. There was a main effect of hemifield, with vertical cues more likely to attract microsaccades than horizontal (χ2 (1) = 30.6, p < 0.001) typically during early CTOAs (χ2 (2) = 6.5, p = 0.039).

**Figure 8:**
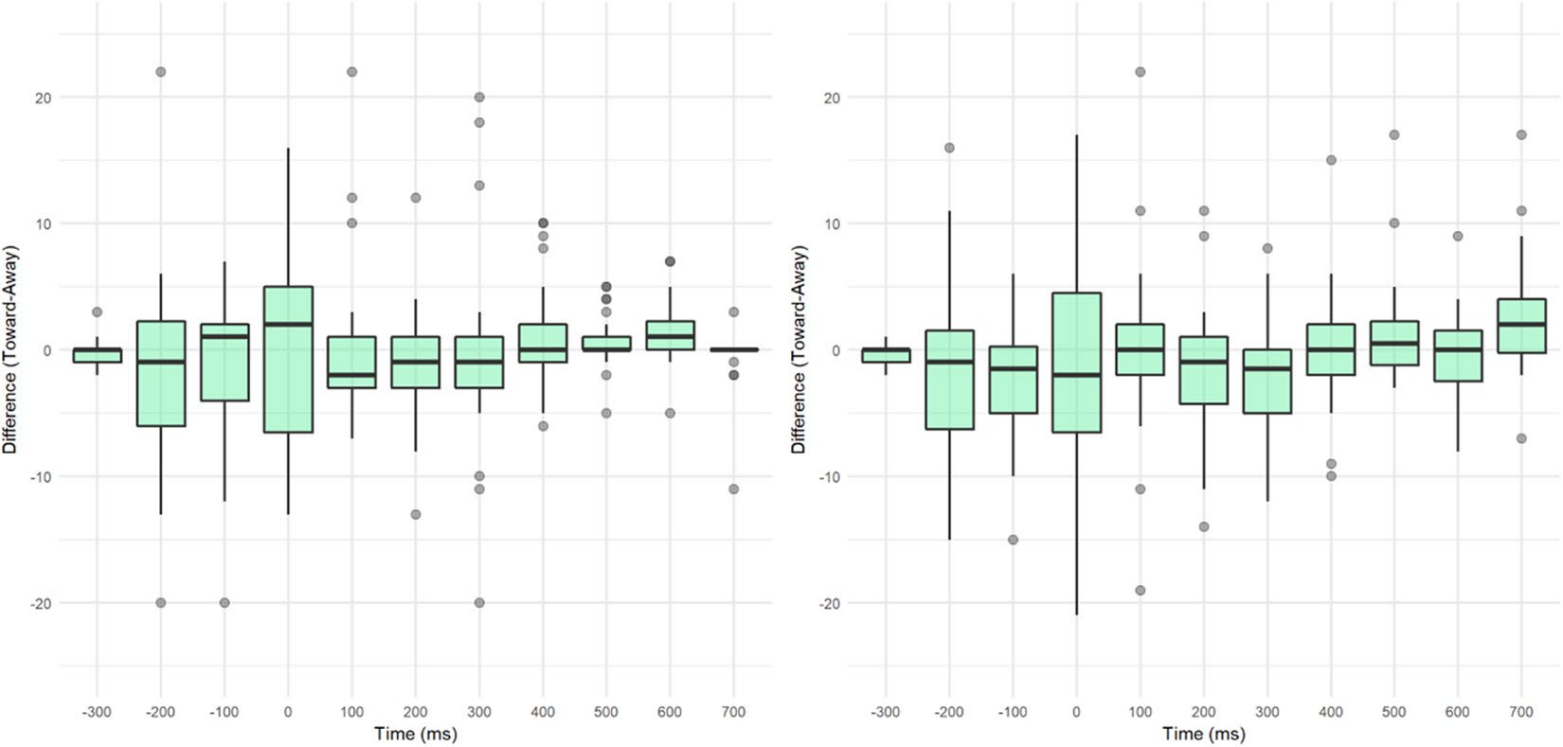
Bias in microsaccadic direction plotted over time (100 ms bins with 0 = cue onset) for (**a**) E1 and (**b**) E2. Difference score is the total number of microsaccades toward the cue minus the total in the opposite direction (binned 45 degrees).

For microsaccadic direction, we again see no change in response to the cue. Our results (Figs 7 and 8) do not show the horizontal bias observed in Laubrock *et al*.^56^, though this is not surprising, since our display had vertical as well as horizontal locations. Also missing in our results is the early bias of microsaccades toward the cue and the later inhibitory bias away from the cue. An ANOVA of the total bias (microsaccades toward the cue minus total opposite the cue) for 100 ms bins before and after the cue shows a small effect of microsaccade time (F (10) = 2.0, p = 0.031), unlike previous research, none of the bins differ significantly from 0 (Fig. 8). Although the changes in observed microsaccade could be related to the lack of facilitation, it’s also possible that the random CTOA influenced the rate and direction of microsaccade activity in response to the cue. Laubrock *et al*.^56^ used a fixed CTOA which allowed participants to build an expectation of event timing, where our random and shorter CTOAs did not.

## Discussion

As expected, the data presented here show robust facilitation of saccadic reaction time at early CTOAs when the timing was predictable within a block (E1); and IOR from the earliest CTOA with no evidence of facilitation when timing was unpredictable (E2). Although attention to exogenous cues is considered automatic^16,80–82^, our study joins recent examples where facilitation of saccadic^36,83^ and manual^36,37^ RT is not observed. Facilitation is not the only measure of attention to an exogenous cue, however, and we observe an impact of the cue on saccadic curvature away from the cued location at early CTOAs but we see similar negative curvature in both experiments instead of the more common positive curvature at short CTOAs. We observe a slight reduction in microsaccade rate at the time of the cue, but this is true for both experiments and much less pronounced than expected. We also observe a change in pupil size in response to the cue in E1 that predicts larger validity effects, but we see no effect of dilation in E2 when there is no coinciding early facilitation. Combined, these results show limited evidence for alternate measures of attention when facilitation is present, and no evidence of attentional capture when facilitation is absent at the cued location. While it is true that the cue was processed to some degree in both experiments, as evidenced by late IOR and curvature away from the orthogonal cued location, we only see evidence for attentional capture with our measures when facilitation was present.

Although we only observe early facilitation in E1, we do observe robust IOR throughout the CTOA range including early CTOAs in E2. Hilchey *et al*.^83^ have suggested a dual account of IOR to explain similar results (their Expt 1: peripheral). Rather than an attentional account, they propose an early oculomotor facilitation caused by residual activity in the intermediate layers of the SC^84–86^. In their first experiment (similar to the method presented here), their lack of faciliatory effect is explained by an early, stronger IOR possibly caused by ‘input’ sensory adaptation. In fact, they only observe oculomotor driven facilitation when the target saccade is signaled by a central arrow. This adaptation is observed neutrally in monkeys and has worked in models of the SC^87,88^ and depends on whether the oculomotor system is inhibited^83^. This account, however would require a second type of ‘output’ IOR for the later CTOAs since sensory adaptation is short lived, measured in a few hundred milliseconds. Given that we do see saccadic facilitation to peripheral cues when the CTOA was predictable within a block(E1) and our lack of interaction between validity and CTOA in E2, we believe a single underlying mechanism is both simpler and more likely.

Saccadic curvature deviations have been explained in terms of population coding theory^89,90^, which states that each neuron in the motor map aligns to a vector, coding movement towards the corresponding location. Eye movements are generated in the direction of the average of the vectors in the oculomotor system. When there are two objects close to each other, the average movement vector would point to an intermediate location. In the case where a single object has to be selected (target), the average movement vector will involve suppression of one vector and hence, a deviation away from the distractor. On the other hand, if the suppression is weak, it results in a deviation towards a distractor. Another interpretation, on the basis of neurophysiological results, states that saccade trajectories are initiated on the basis of weighted average of the corresponding vectors^91^. These results focus, primarily, on the superior colliculus (SC) area of the brain which represents a vector map of the external world and receives inputs from other cortical areas. So, stimulation of particular cells in the SC result in saccades to the location corresponding to the stimulated location. Saccade deviations are seen as competitive interaction within the layers of the SC and a measure of the oculomotor activity. Other explanations of saccadic trajectories involve effect of distractor, temporal and spatial aspects of oculomotor inhibition^92–94^.

In a distractor paradigm, deviation towards the distractor indicates that the distractor activity has not been fully inhibited, deviation away from the distractor represents complete inhibition of distractor activity. This inhibition is further influenced by the strength of the stimulus and the distractor location. Walker, McSorley, and Haggard^48^ suggest that top down inhibitory processes, originating in the frontal eye field (FEF), are applied before stimulus onset when target location is known in advance. In spatially unpredictable target conditions like our paradigm, we should not expect this preparatory inhibition process and should expect a greater possibility of saccades deviating towards the distractor. Pratt, Sekuler and McAuliffe^34^ suggest that the mechanisms for both IOR and facilitation exist very shortly after the cue, but that IOR is typically masked by early facilitation when the bi-phasic pattern is observed^31,33^. Given the deviation away from the uninformative (distractor) cue at early CSOAs in both experiments, we are likely seeing an early suppression of the oculomotor system. This pattern differs from others who find curvature toward a distractor location when the task is unpredictable in nature^47^. Although we observe the curvature diminishing at longer CSOAs, we also do not see the biphasic pattern shift to curvature away from the cue typical of later CTOAs^42^. In addition, for the first experiment in particular shows a dissociation at early CTOAs in processing speed (facilitation of SRT) and saccade programming vector (inhibited) although both diminish at different rates. We believe this adds to the evidence dissociating eye movements and attention^95^ and further limits theories such as the premotor theory of attention^96^.

Although microsaccades are typically reflexive, they can be controlled voluntarily without retinal fading and in some high-acuity tasks they do get suppressed automatically. There seems to be a fixed relation between the micro-saccadic amplitude (degrees) and velocity (degrees/sec) which is seen in a typical linearly increasing pattern called ‘main sequence’^97^, indicating a common generation mechanism for saccades and microsaccades^98–101^. While some have observed more horizontal and vertical than oblique microsaccades^102^, this seems to be task dependent as we observe no effect here with horizontal and vertical targets equally likely. Microsaccades may be absent when the oculomotor system is not engaged^103^, but here we see an absence of microsaccades despite the following saccade to the target. The commonality for microsaccades to an onset might be less about oculomotor engagement, but more as a buildup of anticipation for a saccade that might be absent in the current experiment.

The distinction between pupillary dilation (driven by LC) and PLR (controlled by SC activity) has been highlighted as they refer to different underlying mechanisms^104^. It has been demonstrated that PLR is modulated by higher level cognition or covert attention and should be considered similar to spatial eye movements, seeing that both saccades and PLR have their origins in SC^87,105^. We did see an influence of PLR on our task, in that there was a differential impact on cue validity. In E1, where we also observe facilitation, the change in pupil size as a response to the cue predicted slower SRTs in valid trials as compared to invalid. Given that the PLR can be used to measure covert attention to peripheral onsets, this seems to imply that differences in the degree that a cue is attended influenced our validity effects.

Taken together, these results suggest that these measures reflect different aspects of attention and saccade generation. They also fail to support the hypothesis that attention can be measured independently of facilitation with gaze data. Facilitation of reaction times to exogenous cues has been a key measure of spatial attention since Posner’s original demonstration^80^. As predicted, facilitation was present in E1 and absent in E2, but even when facilitation was present, we did not observe an impact on all of our alternate measures of attention. The pupillary light reflex (PLR) was consistent with SRT results in that greater changes in PLR to the cue matched slower RTs for targets at valid cue locations. Both experiments showed saccadic curvature deviation away from the cued location in orthogonal trials with strongest deviation at early CTOAs and for the vertical hemifield. The only diagnostic results that differed between experiments was a small deviation away from the cue in the second experiment for horizontal saccades that does not appear in E1. Given that we expected a deviation toward the cue to reflect early attention, it is possible that it shows here as a counter to the stronger deviation away caused by oculomotor inhibition. Likewise. microsaccade rate and direction were not seriously impacted by the cue onset despite observing attentional capture as measured by saccadic reaction time. Given that microsaccades may be influenced by top-down attention to the cue, we suggest that relationship is dependent on the task and or attentional set produced by experiment demands. Changes in microsaccade rate might be a sufficient but not necessary repercussion of spatial attention or they may require a level of temporal expectation of a coming saccade that is not present in this design. Saccadic curvature, on the other hand, was influenced by the cued location, but in a manner more typically seen with spatial inhibition brought on by predictable spatial locations.

Although exogenous, ‘bottom-up’ attention is often considered automatic, it can be modulated by top-down attention^93,106–108^. Attentional control settings influenced by experiment design could influence the degree of attentional allocated to the cue and reduce its impact when the likelihood of its usefulness is low. Like others, we find no facilitation of SRTs at the cued location in E2 when the temporal onsets are unpredictable, but we do find evidence that the cue was processed (late IOR) and processed early (early saccadic curvature). We also find a dissociation between SRT and other proposed measured of attention even when facilitation is present. Some of these might be explained by attentional control settings caused by a lack temporal expectation for saccade onset to the target (microsaccades) or separate attentional and motor influences (saccadic curvature). Finally, although we do find an effect of some of our alternate measures, they do not always agree with results of attentional capture as defined by saccadic reaction time. Dissociations between the time course of facilitation and curvature, for example, suggest that they reflect different aspects of the when and where of saccadic programming.
